# Insights into *Octopus maya* cathepsins from metatranscriptome and genome: structure evolutionary relationships and functional role prediction in digestive processes

**DOI:** 10.1242/bio.061778

**Published:** 2025-04-15

**Authors:** Daisy Pineda-Suazo, Francisco Guillén-Chable, Wendy Itzel Escobedo-Hinojosa, Clara E. Galindo-Sánchez, Carlos Rosas

**Affiliations:** ^1^ Unidad Multidisciplinaria de Docencia e Investigación, Facultad de Ciencias UNAM, Puerto de abrigo s/n Sisal, Mpio, Hunucmá, Yucatán 97356, México; ^2^ Unidad de Química en Sisal, Facultad de Química, Universidad Nacional Autónoma de México, Puerto de abrigo s/n, Sisal, Yucatán 97356, México; ^3^ Departamento de Biotecnología Marina, Laboratorio de Genómica Funcional, CICESE, Ensenada, Baja California 22860, México

**Keywords:** *Octopus maya*, Metatranscriptome, Genome, Cathepsin, Digestive physiology

## Abstract

Physiological response to feeding is crucial for various production factors such as feed catabolism and growth. Despite growing significance in red *Octopus maya* aquaculture, large-scale commercial production is limited by not sufficiently knowing their nutritional needs, especially their digestive physiology. Since this species is carnivorous, one of the main feeding aspects is directed to protein digestion, but its enzymatic digestive repertoire has not been studied yet at genomic and transcriptomic levels. This study searched for protease enzymes encoded in *O. maya* genome and expressed in the transcriptome, allowing an initial annotation of genes involved in protein catabolism; 117 amino acid sequences related to ‘octopus digestive enzymes’ were retrieved from 66 available-species’ genomes in the NCBI database, coding for cathepsins, papilins, and metalloproteases. Homology analysis identified 36 homologous sequences from *O. maya* transcriptome and three from its genome. Phylogenetic analysis grouped 37 of 39 sequences into 11 of 14 main clades, offering new insights into the evolutionary relationships and functional roles of these proteases. Phylogenetic and motif analyses resulted in selecting 19 amino acid *O. maya* sequences using multiple sequence alignment that were used to generate three-dimensional protein models. The obtained models revealed a diverse structural architecture among 16 modelled cathepsins; however, their catalytic potential to fully clarify their role in protein hydrolysis and cellular processes remains to be determined. Foundational data provides insights into biochemistry and physiology behind *O. maya* protein digestion. Further complementation of these results with enzymatic characterization of the identified proteases should allow for improved diet formulation in order to foster this species aquaculture.

## Teaser

**Summary:** Comprehensive genomic and transcriptomic analysis of *Octopus maya* proteases reveals evolutionary insights, structural diversity, and potential applications for optimizing protein digestion and sustainable aquaculture practices.

## INTRODUCTION

*Octopus maya* is endemic to the continental shelf off the Yucatán Peninsula, Mexico, supporting one of the most important octopus fisheries worldwide with annual production ranging from 8000 to 20,000 tons (t) (Markaida et al., 2017). Although holobenthic, the species adapts well to captivity and can be maintained on a crab-based lyophilized diet in ponds and tanks (Rosas et al., 2014). However, large-scale commercial production requires better knowledge of its nutritional requirements (Domingues et al., 2007; Lee, 1995), stress (Garrido et al., 2017; Iglesias et al., 2007), and physiological processes involved during digestion. Lack of information about *O. maya* digestive physiology impedes the selection of a diet that can be used at production level.

To design and develop formulated feeds for cephalopods, it is essential to understand their digestive physiology (Andrews et al., 2022). Analysis of digestive enzymatic activity in octopuses has been considered as indicator of their ability to digest different types of food at various life stages (Sánchez et al., 2023; Santiago et al., 2024), breeding conditions (Farías et al., 2016), and their response to environmental factors such as temperature (Uriarte et al., 2016). Octopuses in general have carnivorous eating habits (Cerezo-Valverde et al., 2012; Rosas et al., 2013), so proteolytic enzymes play a key role in their digestive process (Rosas et al., 2011). Thus, the enzymes involved in protein digestion should be highly efficient, with a rapid response capacity to the arrival of food in the digestive tract. Among these proteolytic nature enzymes are trypsin, chymotrypsin (Boucaud-Camou and Boucher-Rodoni, 1983; Martínez et al., 2011), and cathepsins (Barrett and Kirschke, 1981; Gallardo et al., 2017; Ibarra-García et al., 2018).

The proteases trypsin and chymotrypsin are among the most widely studied enzymes in cephalopods (Mancuso et al., 2014; Martínez et al., 2011, 2012; Pereda et al., 2009; Rosas et al., 2011). In contrast, studies on carbohydrases and lipases in cephalopods are limited to *Octopus cyanea* (Boucher-Rodoni, 1973; Omedes et al., 2022), *Eledone cirrosa* [Vitesse de digestion chez les cephalopodes eledone cirrosa (lamarck) et illex illecebrosus (lesueur), 1975], *Octopus vulgaris* (Boucher-Rodoni and Boucaud-Camou, 1987; O'Dor et al., 1984)), *O. maya* (Aguila et al., 2007; Gallardo et al., 2017; Moguel et al., 2010), *Octopus bimaculoides* (Ibarra-García et al., 2018; Solorzano et al., 2009) and *Octopus mimus* (Linares et al., 2015).

Moreover, acidic enzymes as acid phosphatases and cathepsins (B, D, H, and L) were first observed in *O. vulgaris* crop, stomach, and digestive gland (DG) (Morishita et al., 1974). Subsequent studies by Pineda-Suazo et al. (2024), Martínez et al. (2011) and Linares et al. (2015) indicate the presence of acidic enzymes not only in *O. vulgaris* but also in *O. maya* and *O. mimus*. Additionally, these enzymes have been observed in other cephalopod species, such as squid and cuttlefish (Cardenas-Lopez and Haard, 2005, 2009; Perrin et al., 2004), suggesting their relevant role in cephalopod digestive capacity.

In addition, a study in planktonic paralarvae that belong to four cephalopod families: Octopodidae, Bolitaenidae (Octopods), Ommastrephidae and Enoploteuthidae (Oegopsid squids), identified fourteen enzymes involved in digestion and absorption. These enzymes, detected via histochemical methods, include esterases (non-specific), alkaline and acid phosphatases, amylase, acetyl-glycosaminidase, glucuronidase, proteases (non-specific), chymotrypsin, trypsin, aminopeptidase M (AMP M), and dipeptidylaminopeptidases (DAP) I, II, and IV, as well as cathepsin B (Boucaud-Camou and Roper, 1995).

Building on this foundational research, *O. maya* digestive gland and gastric juice enzymes were recently characterized to explore the potential role of cysteine proteinases in their digestion processes (Pineda-Suazo et al., 2024). The study evaluated the effects of selective proteinase inhibitors – leupeptin, pepstatin A, and E64 –which suggested the presence of cathepsins B, H, and L, highlighting their essential role in *O. maya* digestive physiology.

Considering the previous characterization, numerous studies have focused on transcriptomic and genomic analyses in octopuses in recent years, revealing crucial information about their biology and adaptations (Baden et al., 2023; García-Fernández et al., 2019; Juárez et al., 2019; Ramos-Rodríguez et al., 2024). These studies have explored various aspects of *O. vulgaris*, such as the complexity of its central nervous system, where gene expression has been analyzed to better understand functional molecular neurobiology and comparative evolutionary biology (Zhang et al., 2012). Additionally, research has identified genes involved in immune defense and elucidated the molecular basis of octopus tolerance and resistance to coccidiosis (Castellanos-Martínez et al., 2014). In aquaculture contexts, transcriptomic studies have been instrumental in understanding the challenges posed by environmental factors. For instance, *de novo* transcriptome sequencing of octopus early life stages has provided insights into improving culture practices (Prado-Álvarez et al., 2022). Similar studies in *O. vulgaris* have demonstrated the combined effects of diet and temperature on paralarval development, underscoring the need to optimize both environmental and nutritional conditions to enhance aquaculture success (García-Fernández et al., 2019).

Previous studies have identified key genes and protein-protein interaction networks using advanced technologies such as Illumina RNA-Seq, providing a comprehensive view of the biological and adaptive processes in octopuses. For instance, transcriptomic analysis of *Octopus ocellatus* identified genes associated with egg protection in females and the influence of this behavior on the larval immune response, highlighting the complexity of survival mechanisms in these cephalopods (Li et al., 2021). However, to our knowledge, the presence of digestive enzymes in octopus species genome or transcriptome, specifically *O. maya*, has not been evaluated yet.

*O. maya* is a species with significant potential for aquaculture, particularly under controlled laboratory conditions. Moreover, low-scale culture initiatives have been proposed as a means of supporting coastal communities, especially older adults living in the Yucatán Peninsula, where this species is culturally and economically important. These initiatives offer an opportunity to promote sustainable aquaculture practices while improving the livelihoods of these communities.

In the last 20 years, many studies have been performed to find a diet that satisfies octopus nutritional requirements. In that effort, a paste made with crab and squid freeze-dried meal bound with gelatin, vitamins and minerals resulted in a growth rate similar to that obtained when juveniles were fed crab pieces (Martínez et al., 2014). The aforementioned diet was modified and tested on other species, using different ingredients and other forms to prepare the paste and reduce costs (Bastos et al., 2020, 2021; Gallardo et al., 2020; Santiago et al., 2024). However, the main ingredient in all these cases has been crab or squid meat, making the elaborate diet not scalable to production beyond laboratory conditions.

Until now, acidic enzymes are known to be involved and among those enzymes, cathepsins have an essential role in marine protein digestibility in *O. maya* and also in other octopus species. Nevertheless, other studies should be performed to know which other enzymes are involved in octopus digestive processes, so that a re-formulated diet that uses more ecological and economical ingredients could be used for octopus low-scale production in order to benefit people in coastal areas.

To that end, the present study used transcriptomic and genomic data as a reliable first approach to annotate genes encoding proteases involved in *O. maya* food digestion, shedding light at a molecular level on such a process. This knowledge of digestive physiology may contribute to overcoming current obstacles in low-scale production and provide a solid foundation for octopus aquaculture sustainability, ensuring this species' conservation.

## RESULTS

### Phylogenetic analysis

**Fig. 1.**
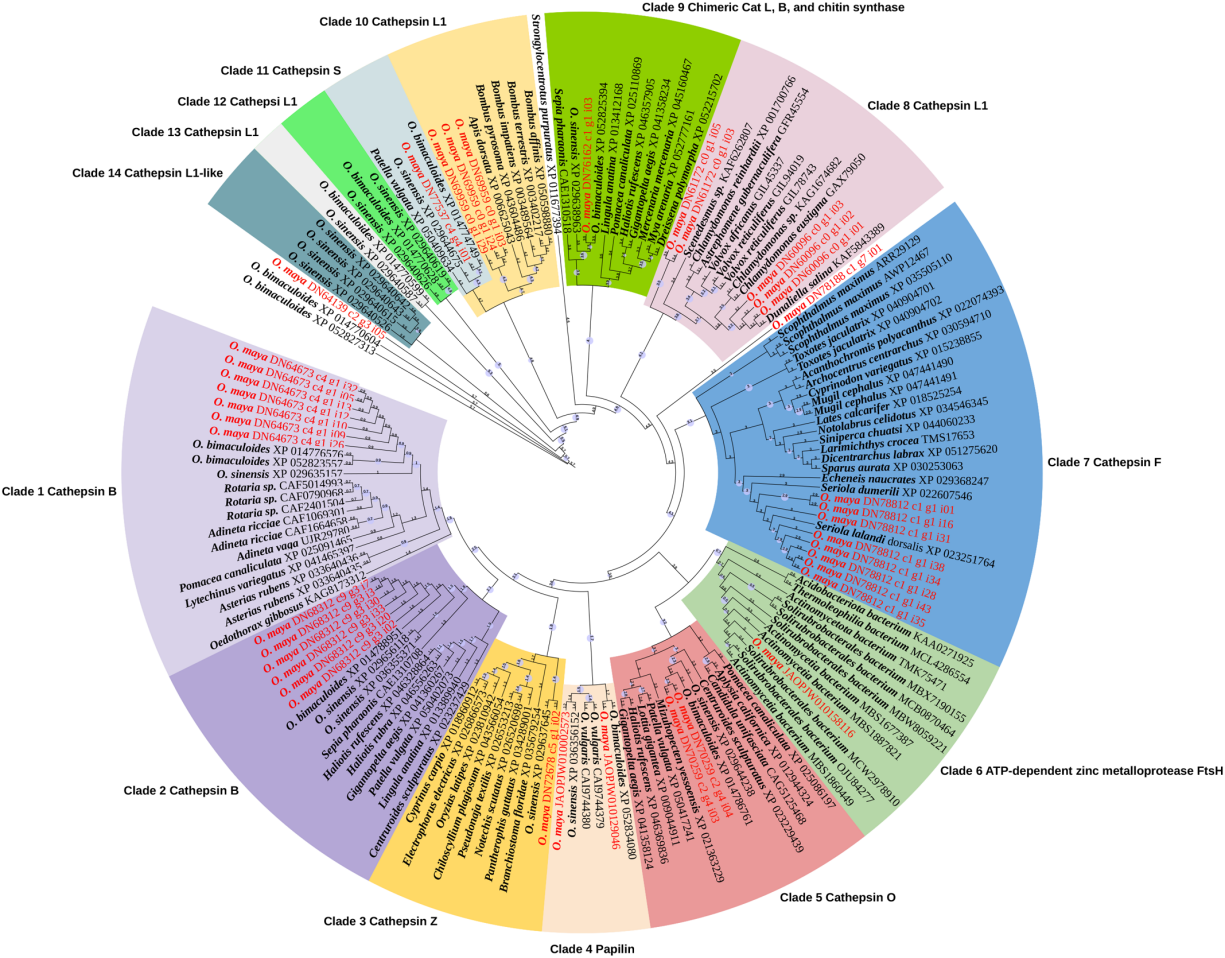
**Phylogenetic analysis (using maximum likelihood) of the gene coding for proteinases retrieved from databases and *O. maya* metatranscriptomics.** Each sequence selected from the NCBI GenBank appears with its accession number. At each node, bootstrap (1000 iterations) values >90 are depicted with a purple circle. Each main clade has a different color. Red typeface indicates *O. maya* enzymes. The number on each tree branch indicates the evolutionary distance in millions of years according to WAG+R6 evolutionary model obtained for the sequences in this analysis.

Phylogenetic analysis identified 117 sequences coding for cathepsins, papilins and metalloproteases in 66 species whose genomes are available in the NCBI database. Moreover, 36 homologous sequences were obtained from *O. maya* transcriptomic database, and three sequences were included that coded for papilins and a metalloprotease from the *O. maya* genome, for a total of 156 sequences (Table S1). Phylogenetic reconstruction of the selected proteinases generated 14 main clades with bootstrap values >90% (Fig. 1). Of the 39 *O. maya* sequences, 37 were within 11 of the 14 main clades and two were outside.

*O. maya* protein sequences were identified by functional family, classifying 12 as members of the first cathepsin group (1 cathepsin Z, 6 cathepsin F, 2 cathepsin L1, and 3 cathepsin B), and two as members of the second group (cathepsin S). Additionally, one counting factor-associated protein D, and 22 enzymes belonging to cysteine proteinases were identified, indicating a robust repertoire of cysteine proteinases (Table S2). Using the term ‘digestive enzyme’ to search in the *O. maya* genome, an ATP-dependent zinc metalloprotease gene *ftsh* and two papilin genes were found.

### Classification of digestive enzymes

To understand the molecular evolution of *O. maya* cathepsins and identify them, the functional motifs analysis was used by visually looking for specific patterns of conserved and divergent motifs. The CGSCWAF motif, conserved across all clades except in clades 4 and 6, indicates the presence of papain family cysteine proteases (Kumar et al., 2013). In clade 4, papilins were aligned, while clade 6 contained ATP-dependent zinc metalloprotease *ftsh* sequences (Table S1). Additionally, the NSW region was identified as a consensus sequence for all cysteine proteases (Yeong Kwon et al., 2001), appearing in specific motifs such as YWLIANSWxxDWGE (cathepsin B-specific; Baig et al., 2002), NSW, and WxVKNSW (cathepsin L-specific; Kumar et al., 2013).

The ERF/WNIN motif, specific to the cathepsin L-like subfamily (Karrer et al., 1993), is absent in cathepsin B-like proteases and modified to ERFNAQ/A in the cathepsin F-like subfamily (Wex et al., 1999, 2002); the protein sequences in clade 5 and 10 contain modified ERF/WNIN motif. The clades 7, 8, 11, 12 and 14 contain the LSEQNLVDC, EXXYPY and WXVKNSW motifs that are cathepsin L-specific (Kumar et al., 2013).

The sequences in clades 8, 10, 12, 13 and 14 were identified as cathepsin L1/L1-like due to the presence of L-specific motifs. However, clade 7 sequences, despite containing L-specific motifs, lacked the ERF/WNIN motif and were classified as cathepsin F based on InterPro analysis. In addition, the enzymes in clade 11 were assigned to the cathepsin S functional family by InterPro.

**Fig. 2.**
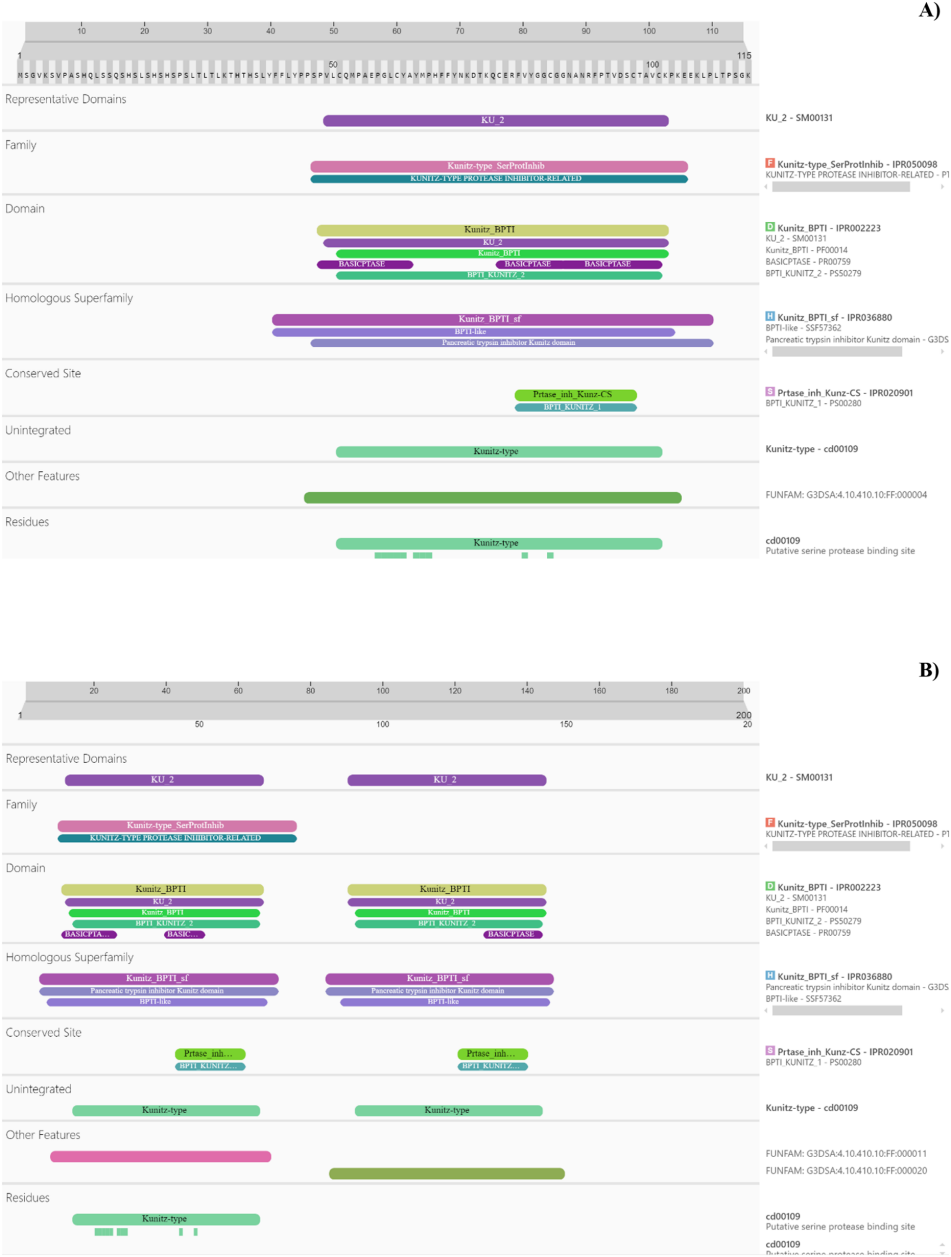
**Analysis of (A) JAOPJW010129046, (B) JAOPJW010002573 protein sequence using InterProScan.** Each colored bar represents a specific domain identified in the protein sequence. The positions of the domains along the protein sequence are indicated by the length scale at the top. The results highlight the presence of catalytic and binding domains, providing a detailed prediction on the possible biological functions of protein XYZ.

In *O. maya* cathepsin B proteins grouped in clade 1, 2 and 9, a variation in three amino acids of the motif YWLIANSWxxDWGE was observed (the variation is highlighted in bold and underlined YWL**I**ANSWxx**D**WG**E**) (Table S1). Additionally, two other conserved functional motifs, CGSCWAF (positions 182–188) and GCNGG (positions 223–227), were identified in clade 1, where cathepsins B are predominantly located. Notably, the GCNGG motif, which is characteristic of all cathepsins B and L, was also detected in clades 9 and 11, grouping cathepsin S and chimeric enzymes, as well as in clade 10 (Karrer et al., 1993). Genome analysis further revealed three genes of interest, two of which belong to the papilin group. The papilin proteins encoded by these genes contain the characteristic pancreatic trypsin inhibitory Kunitz domain (IPR002223) (Fig. 2).

### Structural characteristics and subcellular localization

The 19 *O. maya* amino acid sequences used for homology modeling (Table S3) were analyzed by TMHMM and DeepLoc for the prediction of transmembrane helices and subcellular localization (Table S4 and Table 1). The results from TMHMM and DeepLoc were integrated to provide a comprehensive understanding of the structural and functional properties of the selected *O. maya* proteins. The predicted subcellular localizations were correlated with their phylogenetic placement and motif characteristics, offering insights into their potential roles in cellular and digestive processes. DeepLoc predicted that both papilins sequences and one cathepsin B (DN64673_c4_g1_i5) were localized in the extracellular medium (>99% probability); 1 cathepsin B, 1 cathepsin F, all the cathepsins L1, and the cathepsin S had a >75% probability to be localized in the lysosome/vacuole; and the rest of the nine sequences were localized ubiquitously.

**Table 1.** Subcellular distribution of localization profiles predicted by DeepLoc models of the digestive enzymes analyzed in the present study

Entry ID	Description	Subcellular localization									
		Nucleus	Cytoplasm	Extracellular	Mitochondrion	Cell membrane	Endoplasmic reticulum	Plastid	Golgi apparatus	Lysosome / vacuole	Peroxisome
DN64673_c4_g1_i5	Cathepsin B			99%							
DN64673_c4_g1_i9	Cathepsin B			13%		1%	3%			83%	
DN64673_c4_g1_i13	Cathepsin B	9%	36%	25%	3%	13%	1%		1%	10%	1%
DN72678_c5_g1_i2	Cathepsin Z		1%	47%		1%	6%			45%	
DN78812_c1_g1_i16	Cathepsin F			3%			3%			94%	
DN78812_c1_g1_i38	Cathepsin F		8%	45%	3%	8%	2%	1%	2%	29%	1%
DN78812_c1_g1_i43	Cathepsin F		8%	47%	4%	8%	2%	1%	5%	25%	
DN78812_c1_g1_i35	Cathepsin F	2%	20%	39%	20%	7%	1%	3%	1%	7%	
DN78812_c1_g1_i34	Cathepsin F	2%	11%	36%	19%	8%	2%	2%	4%	14%	1%
DN78812_c1_g1_i28	Cathepsin F	2%	13%	40%	19%	7%	2%	2%	3%	11%	1%
DN60096_c0_g1_i1	Cathepsin L1			5%			9%			86%	
DN61172_c0_g1_i3	Cathepsin L1			21%		2%	2%			75%	
DN61172_c0_g1_i5	Cathepsin L1			20%		2%	2%			76%	
DN69959_c0_g1_i24	Cathepsin L1			5%			3%			91%	
DN77537_c4_g4_i1	Cathepsin S			5%			4%			92%	
DN76162_c1_g1_i3	Counting factor associated protein D			46%			15%			39%	
JAOPJW010129046	Papilin			100%							
JAOPJW010002573	Papilin			100%							
JAOPJW010158116	ATP-dependent zinc metalloprotease ftsh		4%	3%	16%	6%	37%	22%	2%	9%	1%
	**Relative abundance (%)**	1-20	21-40	41-60	61-80	81-100					

Analysis with TMHMM predicted that the TRINITY sequence DN64673_c4_g1_i09 (cathepsin B) contains a single transmembrane region extending from amino acids 25 to 318. The first 60 amino acids exhibit characteristics consistent with a transmembrane region, with approximately 16.5 residues sharing similar properties. The probability of the protein's N-terminus being located intracellularly was calculated at 87.6%. No signal sequence was detected at the N-terminus, suggesting limited membrane transport or signaling involvement. In contrast, other isoforms within this group lacked transmembrane domains and signal sequences, suggesting an extracellular localization and roles distinct from membrane-associated functions. In this study, the *O. maya* cathepsin B group exhibited sequence lengths ranging from 222 to 319 amino acids, with an average molecular weight of 35.2±6.8 kDa.

The isoform TRINITY_DN72678_c5_g1_i02, corresponding to cathepsin Z, displayed a transmembrane region at its N-terminal end along with a potential signal sequence, indicating that it may participate in transport or signaling functions across the membrane in *O. maya*.

The *O. maya* sequences classified as cathepsin F ranged from 345 to 433 amino acids, with an average molecular weight of 43.5±4.6 kDa. None of these isoforms exhibited transmembrane domains or signal sequences at their N-terminal regions, implying that they are unlikely to be membrane-associated. This suggests that cathepsin F isoforms may function primarily in extracellular protein digestion, differing from other cathepsins that interact with the cell membrane or participate in intracellular signaling.

### Homology modeling

**Fig. 3.**
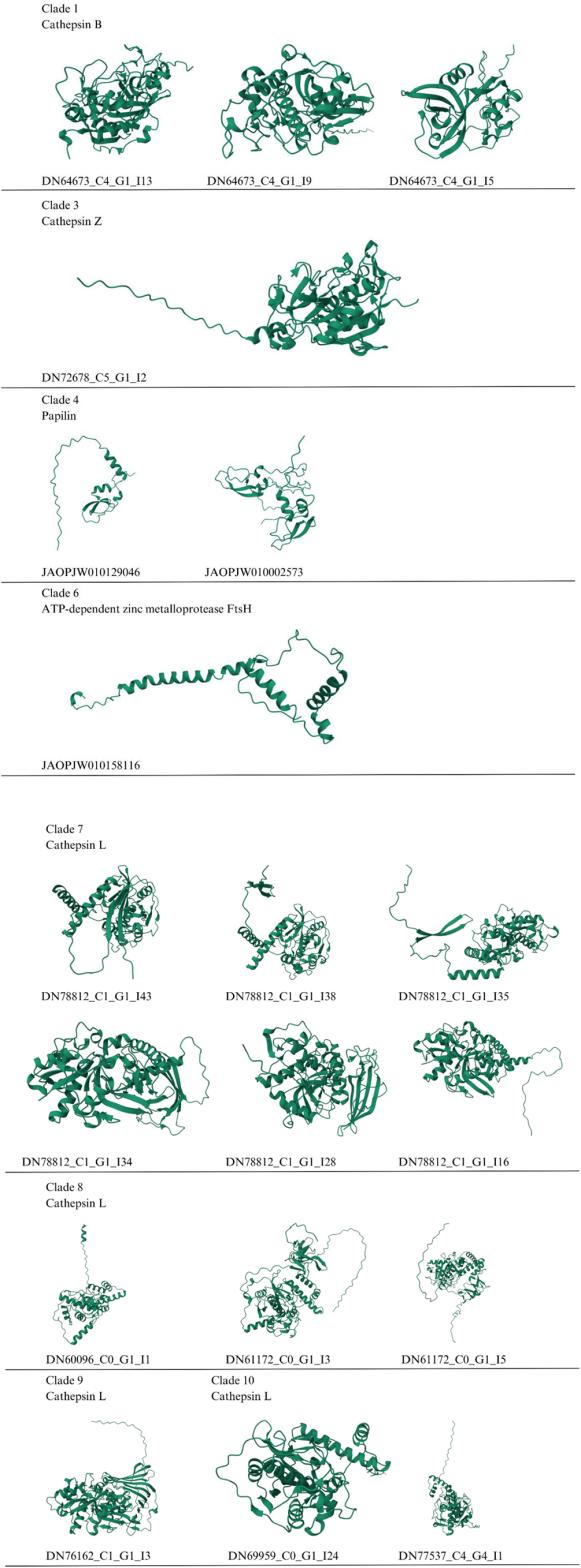
**Three-dimensional AlphaFold2 models of *O. maya* proteins.** Clade numbers as in Fig. 1.

Table S3 shows 19 *O. maya* sequences that were used to generate three-dimensional protein models using AlphaFold2 (Jumper et al., 2021; Varadi et al., 2022) (Fig. 3). The accuracy and quality of the models were improved with GalaxyRefine (Ko et al., 2012; Seok et al., 2021; Shin et al., 2014) and validated by Z-Score on the ProSA-Web server (Sippl, 1993; Wiederstein and Sippl, 2007). An RMSD of 0.238-0.637 Å and poor rotamers at 0-0.9% indicated good superposition and overall similarity between the original and refined structures. Furthermore, the MolProbity scores improved from 0.988-3.418 to 1.154-1.62, indicating higher structural quality of the refined proteins. The occurrence of steric clashes was also significantly reduced, with clash scores of 3.6-12.9 in the refined structures compared to 0.5-29.7 in the original structures (Table S5). The Ramachandran value showed that 98 to 100% of the torsion angles of the peptide bonds were in a ‘favorable’ region (Table S5), indicating a significant improvement in conformation. Overall, the results obtained from GalaxyRefine suggest that the refined protein structure is of high quality and can be used for further docking analysis of the proteins with a selected panel of substrates.

**Fig. 4.**
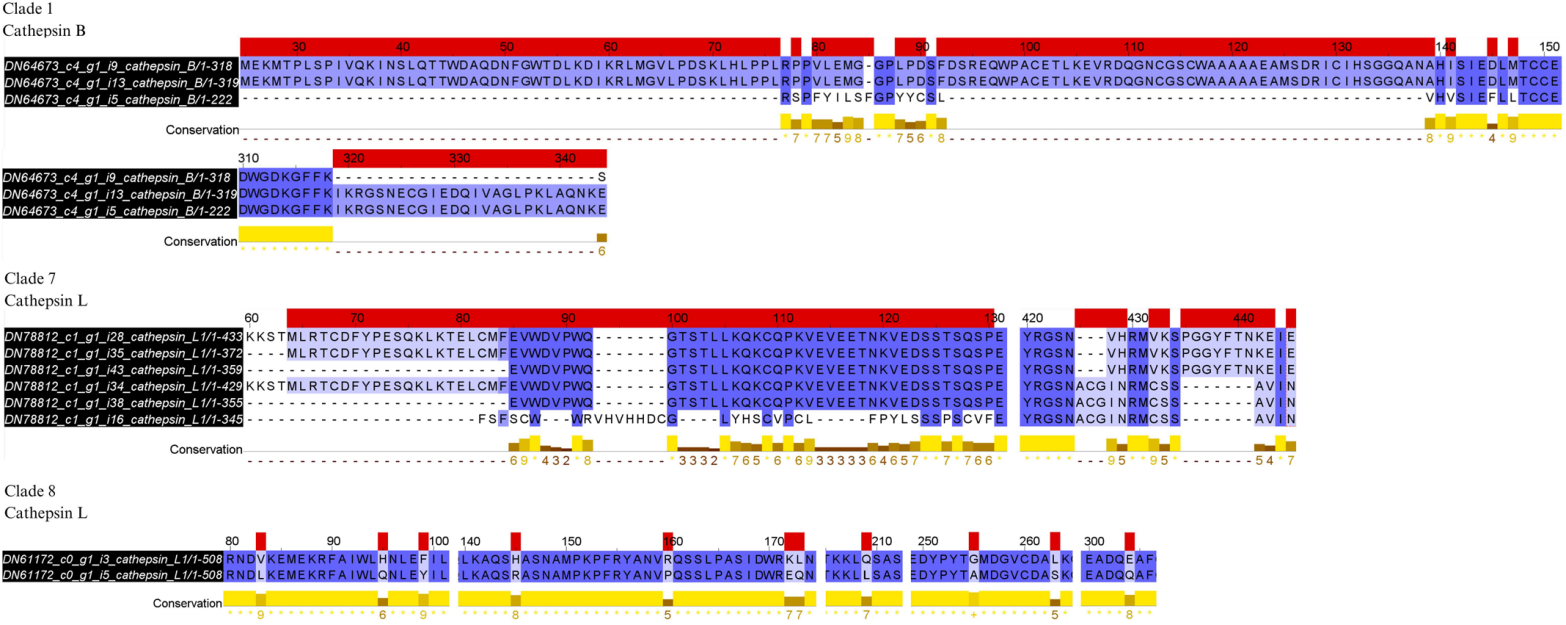
**Multiple sequence alignments of the isoforms of cathepsin B, DN64673, and of cathepsin L, DN78812 and DN61172.** Red areas: differences between sequences within the alignment. Only the regions with differences are displayed; the full alignment is not shown.

Three-dimensional modeling suggested differences in the structure of different protein isoforms from the same gene, which was investigated by performing multiple alignments of the sequences corresponding to the same gene to confirm these differences (Fig. 4). Isoform 5 of cathepsin B, DN64673, differed from isoforms 9 and 13 in nine regions of the amino acid sequence. The six isoforms of the DN78812 sequence differed among themselves in 6 regions. Isoforms 3 and 5 of cathepsin L, DN61172, differed in 11 amino acids.

## DISCUSSION

### Phylogenetic analysis

Cathepsin cysteine proteases, members of the papain (C1) family (Chwieralski et al., 2006), exhibit diverse evolutionary relationships. These proteases are classified into isoforms based on sequence homology, conserved amino acid motifs (cysteine, serine, aspartate), and tissue distribution patterns. Tissue-specific expression divides cathepsins into two major groups: ubiquitously expressed isoforms, including cathepsins B, C, D, F, H, L, O, and Z, and tissue-specific isoforms, such as cathepsins J, K, S, and W (Rawlings and Barrett, 1994a,b; Vidoni et al., 2016). Alternatively, cathepsins are categorized into three subgroups based on sequence homology and conserved motifs: the cathepsin B-like family (B, C, Z), the cathepsin F-like family (F, W), and the cathepsin L-like family (H, L, S, K, V) (Berti and Storer, 1995; Brömme, 2000; Lecaille et al., 2002). These proteases exhibit distinct catalytic activities, functioning as endopeptidases (D, E, F, G, K, L, S, V) or exopeptidases (A, C, X). Notably, some cathepsins, such as B and H, possess dual endo- and exopeptidase activities, highlighting their functional versatility (Barrett and Kirschke, 1981).

Notably, the functional role of cathepsins in invertebrate digestion has been poorly explored, despite their critical importance in protein hydrolysis (Bastos et al., 2020; Cardenas-Lopez and Haard, 2009; Hu and Leung, 2004, 2007; Martínez et al., 2011; Rojo et al., 2010). Cathepsins are central to the digestive process by hydrolyzing complex dietary proteins into absorbable peptides. For example, cathepsin B, known for its dual lysosomal and extracellular proteolytic activity, has been shown to play a key role in protein breakdown in *Litopenaeus vannamei*. Its expression increases post-ingestion and under starvation, reflecting its involvement in both extracellular and intracellular protein hydrolysis (Stephens et al., 2012). Similarly, cathepsin L, another cysteine protease, has been implicated in shrimp cold stress responses, where its downregulation under decreased temperature conditions reduces digestive efficiency (Peng et al., 2016).

Environmental stressors can also influence cathepsin activity, as seen in *Penaeus monodon*, where cathepsin B expression is upregulated in gut, gills, and muscle tissues under low salinity conditions, highlighting its role in managing physiological stress and maintaining digestive efficiency (Shekhar et al., 2013). Additionally, studies in grass shrimp (*Palaemonetes pugio*) have demonstrated the downregulation of cathepsin L under hypoxia, indicating a broader involvement of these proteases in adaptive responses to environmental challenges (Li and Brouwer, 2013).

The connection between cathepsins and prey protein digestion is therefore multifaceted. These enzymes not only hydrolyze dietary proteins but also adapt their activity to physiological and environmental conditions, ensuring optimal digestion and survival. In *O. maya*, the presence of multiple cathepsin types suggests a complex digestive strategy tailored to its carnivorous diet. Understanding how these enzymes function under variable environmental conditions could provide insights into the formulation of diets that maximize digestive efficiency and reduce metabolic stress. This knowledge is particularly relevant for aquaculture, where environmental fluctuations and dietary formulations can significantly impact growth and health.

Previously in Pineda-Suazo et al. (2024), a discrepancy between Martínez et al. (2011) and our results was discussed when cathepsin D inhibitor Pepstatin A was used. Martínez et al. (2011) had suggested the presence of cathepsin D in *O. maya* hepatopancreas and gastric juice, however, no evidence was found supporting the presence of cathepsins D in *O. maya* genome and transcriptome. The use of semi-specific cathepsin D inhibitors or a semi-specific substrate could have led to a wrong interpretation of the enzyme reactions observed by Martínez et al. (2011) regarding cathepsin D.

Instead, cathepsins B, Z, O, F, L1 and, S were found in the present study. Our results agree with the findings of Cárdenas-López and Haard (2005) who suggested that cathepsin L would probably have been detected and inhibited with the use of the specific cathepsin L substrate, Z-PAAFC. Cathepsin L is the most abundant cysteine protease with only endopeptidase activity, which is widely distributed in living organisms, including viruses, bacteria, plants, invertebrates and vertebrates (Barrett and Rawlings, 2001; Berti and Storer, 1995). Cathepsin L is involved in many crucial biological functions in living organisms. Cathepsin L exists in the large vacuole of the B cell, which is the digestive cell and enzyme-secreting cell of the digestive gland of the shrimp *Metapenaeus ensis*, suggesting that it may be required for digestion (Hu and Leung, 2004). Recently, the studies of Hu and Leung demonstrated that *M. ensis* cathepsin L has a food digestion function at both intracellular and extracellular levels, and its digestive model is formulated in the hepatopancreas (Hu and Leung, 2007). Cathepsin L, as a type of parasite proteases, also plays vital roles in nutrient acquisition by catabolizing host proteins into absorbable peptides, excystation, encystment, immune evasion, and invasion of cells and tissues (Dixit et al., 2008; Huang et al., 2009; Ultaigh et al., 2009). Wang and Zhao examined *Cephalochordate amphioxus* cathepsin L developmental expression in embryo, adult, and developing *Branchiostoma belcheri tsingtauense* larvae; the results suggested that cathepsin L participated in proteolytic events (Wang et al., 2008).

Similar to cathepsin L, cathepsin F is probably involved in food digestion; however, to our knowledge only one report exists of cathepsin F participation in digestion although other functions cannot be ruled out, such as the transactivation of other peptidases (Fuzita et al., 2015).

On the other hand, cathepsin S and Z, categorized as lysosomal cysteine proteases (Saikhedkar et al., 2015; Siklos et al., 2015), play crucial roles in the digestive processes of many organisms. Cathepsin S has been purified from carp (*Cyprinus carpio*) and shellfish muscles, and in both cases identified as one of the major proteases that participates in intracellular protein breakdown (Pangkey et al., 2000); hence, it would not be surprising if cathepsin S were part of the repertoire in *O. maya* digestive enzymes. These enzymes (cathepsin S and Z) are synthesized as inactive precursors known as zymogens (Turk et al., 2000, 2012). To become active, cathepsins S and Z require an N-terminal propeptide region removal (Rawlings and Barrett, 1994a,b; Turk, 2001). This propeptide region serves several important functions *in vivo*. Firstly, it aids in the proper folding of newly synthesized enzymes, ensuring they achieve their correct three-dimensional structure. Secondly, it keeps the protease domain inactive until the enzyme reaches the lysosome, preventing premature enzyme activity that could damage cellular components. Lastly, it stabilizes the enzyme against denaturation, protecting it as it is transported to the lysosome where it becomes active and participates in protein degradation (Yamamoto et al., 2002).

In nutritional physiology context, these functions are vital. The proper cathepsin folding and stabilization ensures that the enzymes are fully functional when they reach the lysosome, where they can efficiently break down dietary proteins into amino acids (Agarwal et al., 2020; Naqvi et al., 2022; Yadati et al., 2020). These amino acids are then available for absorption and used by the organism. The enzyme activity regulation through the propeptide region also prevents potential cellular damage from uncontrolled protease activity, maintaining cellular integrity while optimizing nutrient digestion and utilization (Boon et al., 2020; Kitagawa et al., 2021). This precise regulation and activation mechanism highlight the intricate balance required in digestive enzyme function to support effective nutrient assimilation and overall metabolic health.

Finally, cathepsin B appears to be the main enzyme in jumbo squid *Dosidiscus gigas* hepatopancreas (Cardenas-Lopez and Haard, 2005), cuttlefish *Sepia officinalis*, squid *Todarodes pacificus*, and the octopus *O. vulgaris* (Kim et al., 2012; Morishita et al., 1974). The present study suggests its presence in *O. maya* as well.

### Classification of digestive enzymes

By our definition, a motif is a pattern of amino acids that facilitates protein function and protein-protein interactions. These motifs allow classifying many newly identified protein sequences into known families and can be used as tools for predicting protein function (Bork and Koonin, 1996). The motif analysis involves looking for specific patterns in proteins, based on alignments of orthologs, which are proteins with identical functions in different species. In metabolic enzymes, motifs are associated with catalytic functions and thus often readily recognizable. In contrast, structural and regulatory proteins contain more divergent motifs (Bork and Koonin, 1996). However, in some cases, these sequences are so divergent that one can no longer be confident that the motif conservation reflects common ancestry; rather, it may be the result of convergent evolution towards similar binding properties (Doolittle, 1994).

The presence of conserved motifs such as CGSCWAF and NSW across multiple clades underscores the evolutionary conservation of key functional domains in *O. maya* cathepsins. The absence of these motifs in clades 4 and 6 suggests functional divergence, with papilins and ATP-dependent zinc metalloprotease ftsh occupying these clades. These findings highlight the diversification of protease functions within the papain family, reflecting evolutionary adaptations to specific physiological roles.

The identification of clade-specific motifs, such as ERF/WNIN in cathepsin L-like subfamilies and its modification to ERFNAQ/A in cathepsin F-like subfamilies, reveals evolutionary flexibility while maintaining essential catalytic properties. The LSEQNLVDC, EXXYPY, and WXVKNSW motifs, which are hallmarks of cathepsin L, further validate their functional specificity and evolutionary importance.

The classification of enzymes in clades 7 and 11 based on motif absence or modification highlights the importance of integrative analyses combining sequence motifs and functional annotations from tools like InterPro. These findings provide deeper insights into the functional diversity of *O. maya* cathepsins and their evolutionary adaptations, which may be critical for their role in protein hydrolysis and physiological processes. By linking specific motifs to enzyme functionality, this study lays the groundwork for understanding the physiological significance of *O. maya* cathepsins, paving the way for further studies on their catalytic properties and applications in aquaculture.

The *O. maya* cathepsin B-type digestive proteases have the modified motif YWLIANSWxxDWGE, which is believed to be responsible for hemoglobin degradation. This motif has been identified in various phylogenetically diverse helminths with mammalian hemoglobin serving as the selective force for its presence (Baig et al., 2002). As suggested, one- or two-point mutations in the motif region may lead to critical modifications in the proteolytic characteristics of cysteine proteinases, affecting their substrate specificity (Baig et al., 2002). The presence and variation of such motifs in *O. maya* indicate the evolutionary adaptation of their digestive enzymes to efficiently break down specific dietary proteins, such as those found in their prey, thus facilitating effective nutrient absorption and utilization.

The identification of CGSCWAF, GCNGG, and NSW motifs in cathepsins B and L emphasizes the conserved nature of functional regions across cysteine proteases (Yeong Kwon et al., 2001; Karrer et al., 1993). Interestingly, the occurrence of GCNGG in clades associated with cathepsin S and chimeric enzymes suggests potential cross-functional adaptations, extending the functional repertoire of these enzymes.

The discovery of papilin proteins with Kunitz domains underscores their physiological significance in regulating proteolytic activity. Pancreatic trypsin inhibitor regulates the activity of serine proteases trypsin, chymotrypsin, human neutrophil elastase, kallikrein and plasmin and can bind the aspartic protease pepsin and cysteine proteases papain, respectively (García-Fernández et al., 2016). In sea anemones, Kunitz-type protease inhibitors act as neurotoxins and as protease inhibitors to prevent the rapid degradation of the toxins injected into prey animals or predators (Domínguez-Pérez et al., 2013; Honma and Shiomi, 2006; Prentis et al., 2018; Schweitz et al., 1995); their presence in octopuses and related species suggests their importance in physiology and adaptation of these animals also, mainly when some of the digestive enzymes are stored before digestion in the crop, in anticipation of the next meal (Andrews et al., 2022; Gallardo et al., 2020). Precise regulation of proteolytic activity in the gastrointestinal tract is essential for efficient digestion and optimal nutrient absorption. Kunitz-domain papilins could be preventing excessive and uncontrolled digestion, or even autodigestion in the absence of food.

### Structural characteristics and subcellular localization

Cathepsins, a diverse family of lysosomal proteolytic enzymes, play critical roles in both intra- and extracellular processes (Barrett et al., 2012). In this study, 19 *O. maya* amino acid sequences were analyzed for their subcellular localization and structural characteristics, providing valuable insights into their functional roles in cellular and digestive processes.

DeepLoc predictions revealed diverse subcellular localizations for *O. maya* cathepsins, highlighting their functional versatility. For instance, papilin sequences and one cathepsin B isoform (DN64673_c4_g1_i5) were predicted to localize in the extracellular medium, aligning with remodeling and antigen processing processes essential for nutrient assimilation and tissue maintenance (Vidak et al., 2019). Cathepsin B, a proteolytic enzyme with strong hydrolyzing activity and a molecular weight of ∼37 kDa (Kim et al., 2012; Wilson et al., 1998), has been previously detected in the hepatopancreas of octopuses, squids, and cuttlefish (Hurtado et al., 2002; Kim et al., 2012).

The finding that *O. maya* cathepsin B isoforms may act both intracellularly and extracellularly has significant implications for digestion. These enzymes may function within vacuoles in the digestive gland (Martínez et al., 2011) and in gastric juice (Linares et al., 2015), contributing to the species' efficient two-tier digestive system. However, this efficiency could be challenged by the low digestibility of currently formulated diets, as the extracellular localization of most digestive enzymes may limit their ability to break down complex nutrients.

Most cathepsins were predicted to localize within lysosomes or vacuoles (>75% probability), supporting their role in intracellular protein recycling and proteolytic cascades (Ishidoh and Kominami, 2002; Turk et al., 2013). Cathepsin F, with sequence lengths ranging from 345 to 433 amino acids and an average molecular weight of 43.5±4.6 kDa, lacked transmembrane domains or signal sequences. This suggests a specialized role in extracellular protein digestion, differing from the functions of membrane-associated cathepsins (Brix et al., 2008). Studies in bivalves and fish suggest additional roles for cathepsins, such as participation in lysosomal protein digestion or immune response (Gao et al., 2017; Romero et al., 2022). Further studies are needed to elucidate the specific functions of cathepsin F isoforms in *O. maya*.

TMHMM analysis revealed a transmembrane region in cathepsin B (DN64673_c4_g1_i09), supporting its lysosomal role, while cathepsin Z (TRINITY_DN72678_c5_g1_i02) exhibited a transmembrane region and a signal sequence, suggesting involvement in transport or signaling. These findings underscore the evolutionary adaptations of *O. maya* cathepsins to a carnivorous diet, with their distribution across lysosomes, extracellular spaces, and membranes reflecting versatility in protein hydrolysis, nutrient absorption, and cellular maintenance. Proper regulation of cathepsin activity is crucial, as misactivation can lead to cell death or tissue degradation (Chwieralski et al., 2006; Stoka et al., 2016).

In addition to cathepsins, our analysis identified two papilin enzymes, members of the ADAMTS (a disintegrin and metalloproteinase with thrombospondin motifs) family (Kelwick et al., 2015; Porter et al., 2005; Rocks et al., 2008). Papilins are widely distributed across taxa, from nematodes to humans (Ibarra-García et al., 2018). Although knowledge of papilins in Mollusca is limited, studies in *Pinctada maxima* suggest that these enzymes regulate protease functions during development via their Kunitz domains (Gardner et al., 2011).

This study represents the first report of phylogenetic relationships and subcellular localization of papilin homologs in *O. maya*. While papilins are extracellular proteins (Kramerova et al., 2003), their role in *O. maya* digestive physiology remains unclear. Given their potential involvement in gastric juices, these enzymes may function as homologs to digestive proteases. In *Drosophila melanogaster*, papilins facilitate other proteases during development and tissue repair (Kramerova et al., 2000), suggesting that their non-enzymatic activity in *O. maya* could support nutrient digestion or adaptations to food scarcity.

Further research into the role of papilins and cathepsins in *O. maya* digestion could provide critical insights for designing more effective diets and enhancing aquaculture sustainability.

### Homology modeling

Alternative splicing, in which the exons of the same gene are joined in different combinations, results in different but related mRNA transcripts that can be translated to produce different proteins with well-differentiated structures and functions. In some octopus species, RNA editing, an essential resource for gene regulation, adds a layer of complexity to the proteome (Garrett and Rosenthal, 2012); it is affected by temperature, tissue context, genotype, feeding conditions, and age (Garrett and Rosenthal, 2012; Roux et al., 2016). RNA editing has been affected by environmental factors over generations (Zaidan et al., 2018). In ectotherms, temperature changes challenge the integration of physiological function. For example, *Octopus bimaculoides* neural proteome undergoes massive reconfigurations via RNA editing following a temperature challenge (Birk et al., 2023) that could be happening in *O. maya*, a species in which temperature affects reproduction (Juárez et al., 2015; Meza–Buendía et al., 2021) and in which the parental thermal history affects thermal tolerance and energetic plasticity of the offspring (Juárez et al., 2016). It is important to identify factors that regulate digestive functions because the digestive system is responsible not only for nutrient intake but also for defense against pathogenic microbes (Satake et al., 2019). In the wild, the immune condition of *O. maya* changes with the environmental temperatures experienced along its range of distribution (Pascual et al., 2019) and with the concomitant changes in the type of prey (Markaida, 2023). Thus, identifying regulatory factors for digestive functions and immune systems is a key step in understanding *O. maya* life cycle, homeostasis, survival strategy and evolutionary aspects.

## Conclusions

**Fig. 5.**
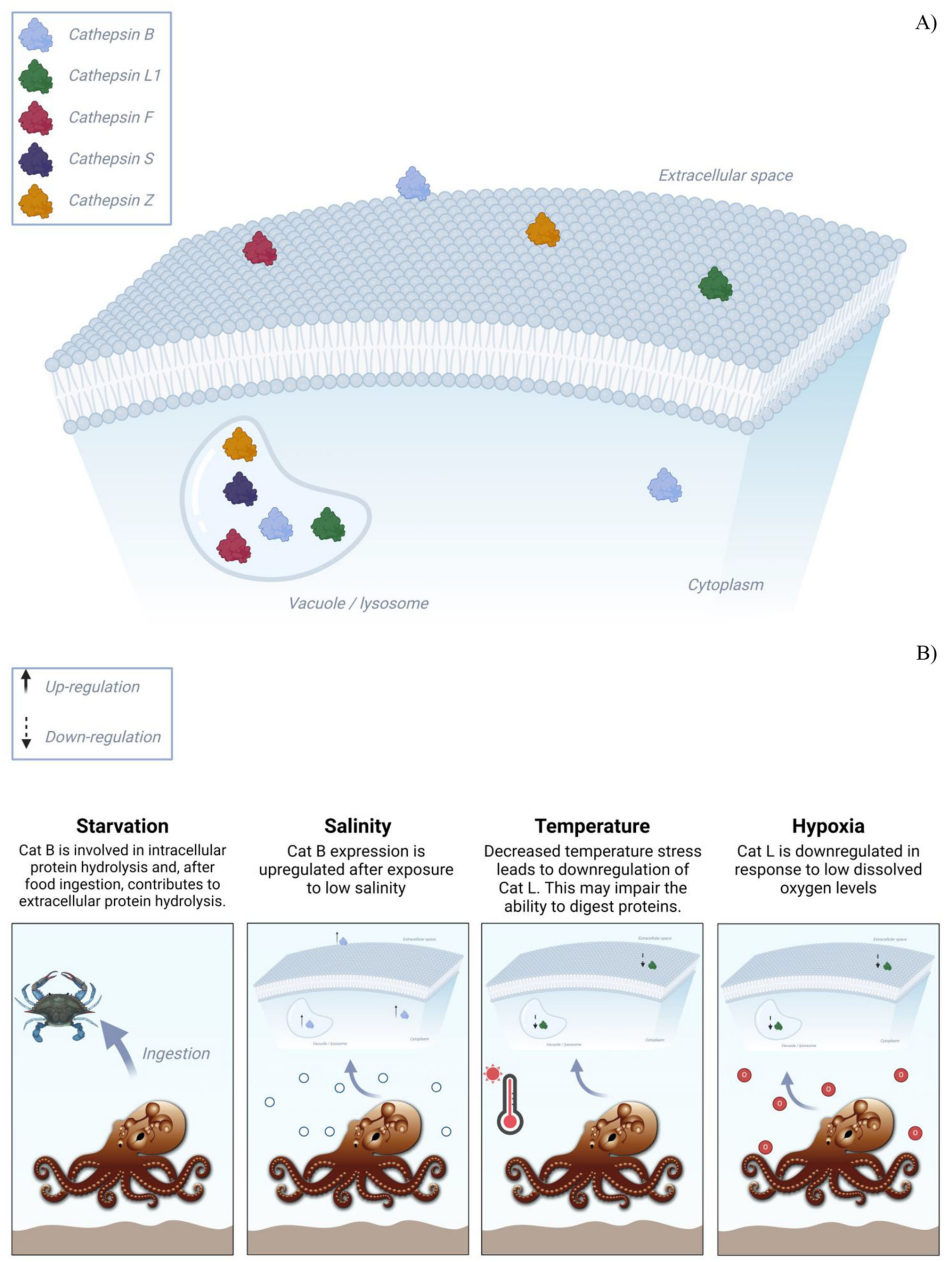
**Schematic representation of the role and regulation of various cathepsins in *O. maya* under different environmental stressors.** (A) Distribution of different cathepsin types (B, L1, F, S, and Z) within the cell, highlighting their involvement in intracellular and extracellular protein degradation. (B) Effects of environmental factors on cathepsin expression.

The present study explored *O. maya* digestive physiology through identifying and characterizing key proteases, particularly cathepsins, using genomic and transcriptomic data. Our phylogenetic and functional analyzes revealed the presence of diverse cathepsin families, highlighting their potential roles in both intracellular and extracellular digestion (Fig. 5A). These findings provide a molecular basis for understanding protein catabolism in *O. maya* and offer insights into the evolutionary relationships between cephalopod digestive enzymes.

The identification of conserved functional motifs, such as CGSCWAF and GCNGG, further suggests a complex regulatory mechanism for these enzymes, which may contribute to their efficiency in breaking down dietary proteins. Additionally, while our research did not directly explore the impact of environmental factors on cathepsin regulation, findings from other species were discussed where such factors (like temperature and salinity) have shown to influence cathepsin expression and digestive function (Fig. 5B). These relationships suggest that similar regulatory mechanisms may exist in *O. maya*, which could be further explored in future studies.

This foundational knowledge of *O. maya* digestive enzymes are crucial for developing optimized artificial diets that can enhance aquaculture production. By improving our understanding of enzyme function and regulation, current challenges can be addressed in large-scale octopus farming and contribute to the sustainable growth of the cephalopod aquaculture industry. Further research should focus on the detailed biochemical characterization of these enzymes to refine dietary formulations and enhance the cultured *O. maya* digestive efficiency.

## MATERIALS AND METHODS

### Digestive enzyme retrieval

For the digestive enzyme retrieval, 156 amino acid sequences were performed using ‘octopus cathepsins’, and ‘digestive enzyme’ as search criteria and then searched their homologies. The sequences were obtained from the National Center for Biotechnology Information (NCBI, https://www.ncbi.nlm.nih.gov/) and *O. maya* genome and meta-transcriptome databases (Table S1). The sequences correspond to 66 organism species, which range from 115 to 599 amino acids in length.

### *O. maya* transcriptomic and genomic enzyme identification

Genomic and transcriptomic data from NCBI regarding *O. maya* genome and transcriptomic data were retrieved. For transcriptomic data with embryonic stages and tissue specific data sets accessible via BioProject PRJNA847320, related to the following tissues: a) systemic b) heart optic lobe and c) oviducal gland.

Whole-genome shotgun data were retrieved from NCBI database with project accession JAOPJW010000000 and the following assembly statistics:

**Table:** 

Total sequence length	2,341,750,473
Total un-gapped length	1,477,916,291
Gaps between scaffolds	0
Number of scaffolds	198,627
Scaffold N50	89,813,523
Scaffold L50	9
Number of contigs	814,385
Contig N50	2361
Contig L50	187,759
Total number of chromosomes and plasmids	0
Number of component sequences (WGS or clone)	198,627

Local annotation of assembled contigs used BLASTX (Altschul, 1997; Altschul and Koonin, 1998). Sequence similarity was sought against UniProtKB/Swiss-Prot (The UniProt Consortium, 2019) protein database with a threshold of e-value <10^−20^ and the following command line: blastx-octopus_maya_contigs.fasta -dbUniprot_database.fasta -out Annotations.txt -*e*value 1*e*–20.

After removing partially matching sequences from the blastx analysis and considering good quality and only matching regions with a percentage of identity between 75-100%, we kept 6467 contigs without any duplicates from the total of annotated as possible genes. Manual curation consisting of handmade research of putative sequences previously identified and annotated from the blastx analysis to identify domains and enzyme families was conducted on InterPro (https://www.ebi.ac.uk/interpro/) on the server online, allow to identify three putative active enzymes corresponding to the following contigs: 1) JAOPJW010158116.1 corresponds to ATP-dependent zinc metalloprotease FtsH 1 (EC 3.4.24.-); 2) JAOPJW010002573.1 corresponds to Kunitz-type serine protease inhibitor 2; and 3) JAOPJW010129046.1 corresponds to another Kunitz-type serine protease inhibitor.

To generate more information about the coding sequences, the *O. maya* genome was retrieved from NCBI with BioSample ID: SAMN27505257 and a *de novo* gene finding was carried out in each contig, with the AUGUSTUS v3.3, a ORF prediction software, confirming homology to active enzymes. *De novo* prediction was conducted with the command line as follows: augustus [parameters] --species=SPECIES queryfilename with *Danio rerio* (zebra fish) as species option. Despite AUGUTUS has limited species options we validated the sequences by the local research on InterPro web repository. Here, we also would like to make a clarification to why we choose *D. rerio*: due to the utilization of a custom database requires and exhaustively constructed position-specific score matrix (pssm) profile file, and we were limited with high computing facilities to develop such profiles files with a more related species we decided to us, the pre-loaded profiles on AUGUSTS as a via to uncover ORF with our computing facilities.

The predicted coding sequences were used to carry out BLAST search on the NCBI database. To assess phylogenetic relationships, the sequences with 85% similarity were downloaded and used for further analyses.

Transcriptomic data from *O. maya* were obtained from NCBI accession number PRJNA544090, with 150 base pair (bp) reads in length and with the SRA files ranging from SRX9923268–SRX9923296 corresponding to the systemic heart, optic lobe, and oviducal gland tissues and from Juárez et al. (2022) publication, four specimens were used for RNA extraction and sequencing. For the data regarding the embryonic stages of *O. maya* under thermal stress transcriptomic data with BioProject number PRJNA847320 and all the SRA files from SRX15643407 to SRX15643424 identifiers were downloaded. The quality of the raw data was verified, and sequences were trimmed and reviewed (see below), which was followed by assembly *de novo* in Trinity v2.4.0 software and the workflow pipeline described by Juárez et al. (2022).

The quality of the raw reads was assessed with FastQC (Simon, 2010). The pre-processing used Trimmomatic v0.36 (Bolger et al., 2014); adapter sequences were removed with TruSeq3-PE from each library and reads <36 bp in length or with an average quality ≤25 (5-base average) were excluded. Additionally, 5-base heads were cropped from each read. The dataset obtained was used for the subsequent analysis. All the pre-processed reads were concatenated into two paired datasets (forward and reverse) and used as input within the *de novo* assembly in Trinity v2.4.0, in a non-strand-specific mode (Haas et al., 2013). To assess the quality of contigs the pre-processed reads were mapped back to the *de novo* assembly. Properly mapped reads and those at >1 read per kilobase of transcript per million mapped reads (RPKM) were the criteria used in trimming out spurious assembled contigs. Additionally, the quality of contigs was inspected with Trinity ExN50_by computing N50 values and the contigs score components. Completeness was inspected against BUSCO (Université de Genève, https://busco.ezlab.org/busco_v4_data.html) sets for Eukaryota and Metazoa (Simão et al., 2015). Both *de novo* transcriptome assembly and all the pre-processed libraries were used as input in a sample-specific expression analysis. All the reads were aligned back against the indexed *de novo* transcriptome assembled with Bowtie2 (Langmead and Salzberg, 2012). Gene and isoform expression calculation levels used the Expectation-Maximization algorithm embedded in Trinity differential expression modules (align_and_estimate_abundance.pl) on a per sample basis. Functional annotation of the contigs used a local BLAST with the NCBI-blast-2.4.0 (Altschul et al., 1990) and Swissprot/Uniprot databases (Apweiler et al., 2004). Hits with an e-value <1*e*-05 were retained. Subsequently, Trinotate v3.0.1 (Bryant et al., 2017) was used to assign the best BLAST result for each protein against SwissProt/UniProt database and predictions for PFAM domains and open reading frames (ORF).

Annotation of assembled contigs again used BLASTX and UniProtKB/Swiss-Prot (see above) with 1*e*^–20^ in the command line. Hits with <50 amino acids were not considered in the search for putative digestive enzymes. AUGUSTUS v3.3 was used to assess homology of each annotated contig to an active enzyme. Active enzyme profiles were constructed in order to carry out the prediction and validation following the command line: msa2prfl.pl fam.aln>fam.prfl and augustus --proteinprofile fam.prfl genome.fa, where fam.aln correspond to the enzyme family alignment and the fam.prfl to the enzyme family profile generated. Then, Augustus was carried out taking account the profile created previously and the genome/transcriptome data as an input for prediction of ORFs. However, we understand the limitations about using profiles of enzyme families due to the lack of any previously information about digestive enzymes on octopus related organisms. And taking into account the computational requirements to carried out the construction of pssm profiles we decided to focus on enzyme families instead of organism related database profile.

### Phylogenetic analysis

All protein sequences were aligned by using MAFFT (v7.511) (Katoh et al., 2005) with L-INS-i algorithm. The resulting alignment was used to select the evolutionary model using IQ-TREE server (Nguyen et al., 2015; Trifinopoulos et al., 2016). The maximum likelihood phylogenetic reconstruction used IQ-TREE, and branch support was assessed by ultrafast bootstrap approximation with 1000 replicates (Hoang et al., 2018). Selection of the best evolutionary model for the analyzed proteins, WAG+R6, was based on Bayesian Information Criterion. The resulting phylogenetic tree was visualized and edited with the iTOL web server (Letunic and Bork, 2019).

### Classification of digestive enzymes

To determine the class of each digestive enzyme obtained from *O. maya* meta-transcriptome, the InterPro database (Blum et al., 2021) was used to analyze the protein domain. Furthermore, selected proteins within each clade were subjected to multiple sequence alignments using PROMALS (Pei et al., 2008), and Motif Enrichment Analysis (MEA) used visual analysis for each alignment.

### Structural characteristics and subcellular localization

The phylogenetic analysis and motif identification described earlier were used as criteria to select 19 *O. maya* amino acid sequences for further structural analysis and homology modeling. The selected sequences were analyzed using the TMHMM algorithm (Hallgren et al., 2022 preprint), a tool designed to predict transmembrane helices based on amino acid properties and sequence alignment. This analysis identified potential transmembrane domains, providing insights into the structural configuration and localization of these proteins.

The DeepLoc model (Thumuluri et al., 2022) was employed to predict the subcellular localization of the selected sequences. This machine learning-based tool categorizes proteins into one or more of the following ten localizations: nucleus, cytoplasm, extracellular space, mitochondrion, cell membrane, endoplasmic reticulum, chloroplast, Golgi apparatus, lysosome/vacuole, and peroxisome. The predictions were cross-referenced with known functional motifs and phylogenetic classifications to ensure consistency.

### Homology modeling

The selection of sequences from *O. maya* for generating three-dimensional protein models was based on the phylogenetic analysis focused on capturing representative sequences from various clades with particular emphasis on the digestive enzymes cathepsin L and cathepsin B (Hu and Leung, 2007). The protein models were generated with AlphaFold2 (Jumper et al., 2021) within the ColabFold framework (Mirdita et al., 2022), employing default parameters and MMseqs2 for homolog searching in the ColabFold database. The accuracy and quality of the modeled structures were enhanced through refinement using the GalaxyRefine server (Heo et al., 2014). The validity of the models was assessed by calculating Z-scores using the ProSA-Web server (Wiederstein and Sippl, 2007). The three-dimensional structures were visualized and analyzed with Mol* 3D Viewer (https://www.rcsb.org/3d-view) (Sehnal et al., 2021).

## Supplementary Material

10.1242/biolopen.061778_sup1 Supplementary information
