# Supplementary material for: Insights into Octopus maya cathepsins from metatranscriptome and genome: structure evolutionary relationships and functional role prediction in digestive processes
Source: Biol Open. 2025 Apr 15;14(4):bio061778. doi: 10.1242/bio.061778 (PMC12032550; doi:10.1242/bio.061778)
Supplement: Supplementary information [file biolopen-14-061778-s1.pdf]

Table S1. Proteins used for phylogenetic analysis.

Clade	Sequence	Species	Description
	TRINITY DN64673 c4 g1 i32	*Octopus maya*	Cathepsin B
	TRINITY DN64673 c4 g1 05	*Octopus maya*	Cathepsin B
	TRINITY DN64673 c4 g1 i13	*Octopus maya*	Cathepsin B
	TRINITY DN64673 c4 g1 i12	*Octopus maya*	Cathepsin B
	TRINITY DN64673 c4 g1 i10	*Octopus maya*	Cathepsin B
1	TRINITY DN64673 c4 g1 i09	*Octopus maya*	Cathepsin B
	TRINITY DN64673 c4 g1 i26	*Octopus maya*	Cathepsin B
	XP_014776576	*Octopus bimaculoides*	Cathepsin B
	XP_052823557	*Octopus bimaculoides*	Cathepsin B isoform X1
	XP_029635157	*Octopus sinensis*	Cathepsin B
	CAF5014993	*Rotaria* sp.	Unnamed protein product

	CAF0790968	*Rotaria* sp.	Unnamed protein product
	CAF2401504	*Rotaria* sp.	Unnamed protein product
	CAF1069301	*Adineta ricciae*	Unnamed protein product
	CAF1664658	*Adineta ricciae*	Unnamed protein product
	UJR29780	*Adineta vaga*	Hypothetical protein
	XP_025091465	*Pomacea canaliculata*	Cathepsin B-like
	XP_041465397	*Lytechinus variegatus*	Cathepsin B-like
	XP_033640436	*Asterias rubens*	Cathepsin B-like isoform X2
	XP_033640435	*Asterias rubens*	Cathepsin B-like isoform X1
	KAG8173312	*Oedothorax gibbosus*	Hypothetical protein
2	TRINITY DN68312 c9 g3 i07	*Octopus maya*	Unnamed protein product
	TRINITY DN68312 c9 g3 i03	*Octopus maya*	Unnamed protein product
	TRINITY DN68312 c9 g3 i30	*Octopus maya*	Unnamed protein product
	TRINITY DN68312 c9 g3 i33	*Octopus maya*	Unnamed protein product

TRINITY DN68312 c9 g3 i20	*Octopus maya*	Unnamed protein product
TRINITY DN68312 c9 g3 i02	*Octopus maya*	Unnamed protein product
XP_014788951	*Octopus bimaculoides*	Tubulointerstitial nephritis antigen-like
XP_029656118	*Octopus sinensis*	Uncharacterized peptidase C1-like protein
XP_036355371	*Octopus sinensis*	Tubulointerstitial nephritis antigen-like isoform
CAE1310708	*Sepia pharaonis*	Tubulointerstitial nephritis antigen-like
XP_046328864	*Haliotis rufescens*	Uncharacterized peptidase C1-like protein
XP_046556263	*Haliotis rubra*	Uncharacterized peptidase C1-like protein
XP_041360267	*Gigantopelta aegis*	Uncharacterized peptidase C1-like protein
XP_050402073	*Patella vulgata*	Uncharacterized peptidase C1-like protein
XP_013389940	*Lingula anatina*	Uncharacterized peptidase C1-like protein
XP_023237429	*Centruroides sculpturatus*	Uncharacterized peptidase C1-like protein

3	XP_018960912	*Cyprinus carpio*	Cathepsin Z
	XP_026866573	*Electrophorus electricus*	Cathepsin Z

Biology Open • Supplementary information

	XP_023810942	*Oryzias latipes*	Cathepsin Z
	XP_043566054	*Chiloscyllium plagiosum*	Cathepsin Z-like isoform X2
	XP_026553213	*Pseudonaja textilis*	Cathepsin Z isoform X2
	XP_026520698	*Notechis scutatus*	Cathepsin Z
	XP_034289001	*Pantherophis guttatus*	Cathepsin Z-like
	XP_035679254	*Branchiostoma floridae*	Cathepsin Z-like isoform X1
	XP_029637645	*Octopus sinensis*	Cathepsin Z
	TRINITY DN72678 c5 g1 i02	*Octopus maya*	Cathepsin Z
4	JAOPJW010002573	*Octopus maya*	Papilin
	XP_036356152	*Octopus sinensis*	Papilin
	CAI9744380	*Octopus vulgaris*	Papilin
	CAI9744379	*Octopus vulgaris*	Papilin
	JAOPJW010129046	*Octopus maya*	Papilin
	XP_052834080	*Octopus bimaculoides*	Papilin

Biology Open • Supplementary information

	XP_041358124	*Gigantopelta aegis*	Cathepsin O-like isoform X1
	XP_046369836	*Haliotis rufescens*	Cathepsin O-like
	XP_009044911	*Lottia gigantea*	Hypothetical protein
	XP_050417241	*Patella vulgata*	Cathepsin O-like
	XP_021363229	*Mizuhopecten yessoensis*	Cathepsin O-like
	TRINITY DN70259 c2 g4 i03	*Octopus maya*	Cathepsin O
5	TRINITY DN70259 c2 g4 i04	*Octopus maya*	Cathepsin O
	XP_014786761	*Octopus bimaculoides*	Cathepsin O
	XP_029644238	*Octopus sinensis*	Cathepsin O
	XP_023229439	*Centruroides sculpturatus*	Cathepsin O-like
	CAG5125468	*Candidula unifasciata*	Unnamed protein product
	XP_012944324	*Aplysia californica*	Cathepsin O
	XP_025086197	*Pomacea canaliculata*	Cathepsin O-like
6	MBS1860449	*Actinomycetia bacterium*	AAA familyatpase

Biology Open • Supplementary information

	OJU84277	*Solirubrobacterales bacterium*	Cell divisionproteinftsh
	MCW2978910	*Solirubrobacterales bacterium*	ATP-dependent metallopeptidase ftsh/Yme1/Tma family protein
	JAOPJW010158116	*Octopus maya*	ATP-dependent zinc metalloprotease ftsh
	MBS1887821	*Actinomycetia bacterium*	ATP-dependent zinc metalloprotease ftsh
	MBS1677387	*Actinomycetia bacterium*	ATP-dependent zinc metalloprotease ftsh
	MBW8059221	*Solirubrobacterales bacterium*	ATP-dependent metallopeptidase ftsh/Yme1/Tma family protein
	MCB0870464	*Solirubrobacterales bacterium*	ATP-dependent metallopeptidase ftsh/Yme1/Tma family protein
	MBX7190155	*Solirubrobacterales bacterium*	ATP-dependent zinc metalloprotease ftsh
	TMK75471	*Actinomycetota bacterium*	ATP-dependent metallopeptidase ftsh/Yme1/Tma family protein
	MCL4286554	*Thermoleophilia bacterium*	ATP-dependent zinc metalloprotease ftsh
	KAA0271925	*Acidobacteriota bacterium*	ATP-dependent metallopeptidase ftsh/Yme1/Tma family protein
	TRINITY DN78812 c1 g1 i35	*Octopus maya*	Cathepsin L1
7	TRINITY DN78812 c1 g1 i43	*Octopus maya*	Cathepsin L1
	TRINITY DN78812 c1 g1 i28	*Octopus maya*	Cathepsin L1

Biology Open • Supplementary information

TRINITY DN78812 c1 g1 i34	*Octopus maya*	Cathepsin L1
TRINITY DN78812 c1 g1 i38	*Octopus maya*	Cathepsin L1
XP_023251764	*Seriola lalandi dorsalis*	Cathepsin L-like
TRINITY DN78812 c1 g1 i31	*Octopus maya*	Cathepsin L1
TRINITY DN78812 c1 g1 i16	*Octopus maya*	Cathepsin L1
TRINITY DN78812 c1 g1 i01	*Octopus maya*	Cathepsin L1
XP_022607546	*Seriola dumerili*	Cathepsin L-like
XP_029368247	*Echeneis naucrates*	Cathepsin F-like
XP_030253063	*Sparus aurata*	Cathepsin L-like
XP_051275620	*Dicentrarchus labrax*	Cathepsin F
TMS17653	*Larimichthys crocea*	Cathepsin F
XP_044060233	*Siniperca chuatsi*	Cathepsin F
XP_034546345	*Notolabrus celidotus*	Cathepsin F
XP_018525254	*Lates calcarifer*	Cathepsin F

	XP_047441491	*Mugil cephalus*	Cathepsin F isoform X2
	XP_047441490	*Mugil cephalus*	Cathepsin F isoform X1
	XP_015238855	*Cyprinodon variegatus*	PREDICTED: cathepsin W
	XP_030594710	*Archocentrus centrarchus*	Cathepsin F-like
	XP_022074393	*Acanthochromis polyacanthus*	Cathepsin F
	XP_040904702	*Toxotes jaculatrix*	Cathepsin F isoform X2
	XP_040904701	*Toxotes jaculatrix*	Cathepsin F isoform X1
	XP_035505110	*Scophthalmus maximus*	Cathepsin F
	AWP12467	*Scophthalmus maximus*	Cathepsin F
	ARR29129	*Scophthalmus maximus*	Cathepsin F
	TRINITY DN78188 c1 g7 i01	*Octopus maya*	Cathepsin L1
	KAF5843389	*Dunaliella salina*	Xylemcysteine proteinase 2-like
8	TRINITY DN60096 c0 g1 i01	*Octopus maya*	Cathepsin L1-like
	TRINITY DN60096 c0 g1 i02	*Octopus maya*	Cathepsin L1

Biology Open • Supplementary information

	TRINITY DN60096 c0 g1 i03	*Octopus maya*	Cathepsin L1
	GAX79050	*Chlamydomonas eustigma*	Hypothetical protein
	KAG1674682	*Chlamydomonas* sp.	Hypothetical protein
	GIL78743	*Volvox reticuliferus*	Hypothetical protein
	GIL94019	*Volvox reticuliferus*	Hypothetical protein
	GIL45337	*Volvox africanus*	Hypothetical protein
	GFR45554	*Astrephomene gubernaculifera*	Hypothetical protein
	XP_001700766	*Chlamydomonas reinhardtii*	Uncharacterized protein
	KAF6262807	*Scenedesmus* sp.	Hypothetical protein
	TRINITY DN61172 c0 g1 i03	*Octopus maya*	Cathepsin L1
	TRINITY DN61172 c0 g1 i05	*Octopus maya*	Cathepsin L1
	XP_052215702	*Dreissena polymorpha*	Digestive cysteine proteinase 1-like
9	XP_052777161	*Mya arenaria*	Digestive cysteine proteinase 1-like
	XP_045160467	*Mercenaria mercenaria*	Digestive cysteine proteinase 1-like

Biology Open • Supplementary information

XP_041358234	*Gigantopelta aegis*	Digestive cysteine proteinase 2-like
XP_046357905	*Haliotis rufescens*	Digestive cysteine proteinase 1-like
XP_025110869	*Pomacea canaliculata*	Digestive cysteine proteinase 2-like
XP_013412168	*Lingula anatina*	Digestive cysteine proteinase 2-like
XP_052825394	*Octopus bimaculoides*	Digestive cysteine proteinase 2
TRINITY DN76162 c1 g1 i03	*Octopus maya*	Cathepsin L1
XP_029638963	*Octopus sinensis*	Digestive cysteine proteinase 1
CAE1310518	*Sepia pharaonis*	Cysteine proteinase
XP_011677394	*Strongylocentrotus purpuratus*	Cathepsin L1
XP_050598880	*Bombus affinis*	Cathepsin L
XP_003402217	*Bombus terrestris*	Cathepsin L
XP_003489564	*Bombus impatiens*	Cathepsin L
XP_043604486	*Bombus pyrosoma*	Cathepsin L
XP_006625043	*Apis dorsata*	Cathepsin L1

Biology Open • Supplementary information

	TRINITY DN69959 c0 g1 i03	*Octopus maya*	Cathepsin L1
	TRINITY DN69959 c0 g1 i24	*Octopus maya*	Cathepsin L1
	TRINITY DN69959 c0 g1 i29	*Octopus maya*	Cathepsin L1
11	XP_014774749	*Octopus bimaculoides*	Procathepsin L
	TRINITY DN77537 c4 g4 i01	*Octopus maya*	Procathepsin L
	XP_029644675	*Octopus sinensis*	Cathepsin L1
	XP_050409634	*Patella vulgata*	Procathepsin L-like
12	XP_029640619	*Octopus sinensis*	Cathepsin L1-like
	XP_014770628	*Octopus bimaculoides*	Procathepsin L
	XP_029640626	*Octopus sinensis*	Cathepsin L1
13	XP_014770599	*Octopus bimaculoides*	Procathepsin L
	XP_029640587	*Octopus sinensis*	Cathepsin L1
14	XP_029640642	*Octopus sinensis*	Cathepsin L1 isoform X1
	XP_029640643	*Octopus sinensis*	Cathepsin L1 isoform X2

Biology Open • Supplementary information

XP_029640615	*Octopus sinensis*	Cathepsin L1-like
XP_029640526	*Octopus sinensis*	Cathepsin L1-like isoform X1
TRINITY DN64139 c2 g3 i05	*Octopus maya*	Procathepsin L
XP_014770604	*Octopus bimaculoides*	Procathepsin L
XP_052827313	*Octopus bimaculoides*	Procathepsin L isoform X2

Biology Open • Supplementary information

Table S2. Classification of *Octopus maya* digestive enzymes: **aa**, amino acids; **FunFam**, functional family

Sequence	Lengthaa	FunFam	Family	Domain
TRINITY DN64673 c4 g1 i09	318	Cysteine proteinases	Peptidase C1A	Peptidase C1A, propeptide
				Peptidase C1A, papain C-terminal
TRINITY DN64673 c4 g1 i12	343	Cysteine proteinases	Peptidase C1A	Peptidase C1A, papain C-terminal
				Peptidase C1A, propeptide
TRINITY DN64673 c4 g1 i10	343	31: Cathepsin B	Peptidase C1A	Peptidase C1A, papain C-terminal
				Peptidase C1A, propeptide
TRINITY DN64673 c4 g1 i32	411	Cysteine proteinases	Peptidase C1A	Peptidase C1A, papain C-terminal
				Peptidase C1A, propeptide
TRINITY DN64673 c4 g1 i05	222	Cysteine proteinases	Peptidase C1A	Peptidase C1A, papain C-terminal
		Peptidase C1A CathepsinB		
TRINITY DN64673 c4 g1 i13	319	31: Cathepsin B	Peptidase C1A	Peptidase C1A, papain C-terminal
				Peptidase C1A, propeptide
TRINITY DN64673 c4 g1 i26	261	31: Cathepsin B	Peptidase C1A	Peptidase C1A, papain C-terminal
TRINITY DN68312 c9 g3 i02	435	Cysteine proteinases	Peptidase C1A	Peptidase C1A, papain C-terminal
				Somatomedin B domain
TRINITY DN68312 c9 g3 i20	501	Cysteine proteinases	Peptidase C1A	Somatomedin B domain
				Peptidase C1A, papain C-terminal

Biology Open • Supplementary information

TRINITY DN68312 c9 g3 i30	501	Cysteine proteinases	Peptidase C1A	Somatomedin B domain
				Peptidase C1A, papain C-terminal
TRINITY DN68312 c9 g3 i03	501	Cysteine proteinases	Peptidase C1A	Somatomedin B domain
				Peptidase C1A, papain C-terminal
TRINITY DN68312 c9 g3 i07	501	Cysteine proteinases	Peptidase C1A	Somatomedin B domain
				Peptidase C1A, papain C-terminal
TRINITY DN68312 c9 g3 133	501	Cysteine proteinases	Peptidase C1A	Somatomedin B domain
				Peptidase C1A, papain C-terminal
TRINITY DN72678 c5 g1 i02	307	60: Cathepsin Z	Cathepsin Z	Peptidase C1A, papain C-terminal
			Peptidase C1A	
TRINITY DN70259 c2 g4 i03	349	Cysteine proteinases	Peptidase C1A	Peptidase C1A, papain C-terminal
				Papain-like cysteine endopeptidase
				Cathepsin propeptide inhibitor domain (I29)
TRINITY DN70259 c2 g4 i04	309	Cysteine proteinases	Peptidase C1A	Papain-like cysteine endopeptidase
				Peptidase C1A, papain C-terminal
				Cathepsin propeptide inhibitor domain (I29)
TRINITY DN78812 c1 g1 i28	433	Cysteine proteinases	Peptidase C1A	Cathepsin propeptide inhibitor domain (I29)
				Papain-like cysteine endopeptidase
				Cystatin domain
				Peptidase C1A, papain C-terminal

Biology Open • Supplementary information

TRINITY DN78812 c1 g1 i34	429	50: Cathepsin F	Peptidase C1A	Papain-like cysteine endopeptidase
				Cathepsin propeptide inhibitor domain (I29)
				Peptidase C1A, papain C-terminal
				Cystatin domain
TRINITY DN78812 c1 g1 i31	435	50: Cathepsin F	Peptidase C1A	Papain-like cysteine endopeptidase
				Cathepsin propeptide inhibitor domain (I29)
				Peptidase C1A, papain C-terminal
				Cystatin domain
TRINITY DN78812 c1 g1 i01	345	50: Cathepsin F	Peptidase C1A	Papain-like cysteine endopeptidase
				Peptidase C1A, papain C-terminal
				Cathepsin propeptide inhibitor domain (I29)
TRINITY DN78812 c1 g1 135	372	Cysteine proteinases	Peptidase C1A	Cathepsin propeptide inhibitor domain (I29)
				Peptidase C1A, papain C-terminal
				Papain-like cysteine endopeptidase
TRINITY DN78812 c1 g1 i16	345	50: Cathepsin F	Peptidase C1A	Papain-like cysteine endopeptidase
				Cathepsin propeptide inhibitor domain (I29)
				Peptidase C1A, papain C-terminal
TRINITY DN78812 c1 g1 i38	355	50: Cathepsin F	Peptidase C1A	Cathepsin propeptide inhibitor domain (I29)
				Peptidase C1A, papain C-terminal
				Papain-like cysteine endopeptidase

TRINITY DN78812 c1 g1 i43	359	50: Cathepsin F	Peptidase C1A	Peptidase C1A, papain C-terminal
				Cathepsin propeptide inhibitor domain (I29)
				Papain-like cysteine endopeptidase
TRINITY DN78188 c1 g7 i01	340	Cysteine proteinases	Peptidase C1A	Cathepsin propeptide inhibitor domain (I29)
				Peptidase C1A, papain C-terminal
				Papain-like cysteine endopeptidase
TRINITY DN60096 c0 g1 i03	476	Cysteine proteinases	Peptidase C1A	Granulin
		332:Cathepsin L1		Cathepsin propeptide inhibitor domain (I29)
				Peptidase C1A, papain C-terminal
				Papain-like cysteine endopeptidase
TRINITY DN60096 c0 g1 102	476	Cysteine proteinases	Peptidase C1A	Peptidase C1A, papain C-terminal
				Granulin
				Cathepsin propeptide inhibitor domain (I29)
				Papain-like cysteine endopeptidase
TRINITY DN60096 c0 g1 i01	293	Cysteine proteinases	Peptidase C1A	Peptidase C1A, papain C-terminal
				Papain-like cysteine endopeptidase
				Cathepsin propeptide inhibitor domain (I29)
TRINITY DN61172 c0 g1 i03	546	Cysteine proteinases	Peptidase C1A	Papain-like cysteine endopeptidase
				Cathepsin propeptide inhibitor domain (I29)
				Peptidase C1A, papain C-terminal

Biology Open • Supplementary information

TRINITY DN61172 c0 g1 i05	508	Cysteine proteinases	Peptidase C1A	Cathepsin propeptide inhibitor domain (I29)
				Papain-like cysteine endopeptidase
				Granulin
				Peptidase C1A, papain C-terminal
TRINITY DN76162 c1 g1 i03	546	87: Counting factor associated protein D	Peptidase C1A	Papain-like cysteine endopeptidase
				Cathepsin propeptide inhibitor domain (I29)
				Peptidase C1A, papain C-terminal
TRINITY DN77537 c4 g4 i01	338	6: Cathepsin S	Peptidase C1A	Papain-like cysteine endopeptidase
				Peptidase C1A, papain C-terminal
				Cathepsin propeptide inhibitor domain (I29)
TRINITY DN69959 c0 g1 i03	508	Cysteine proteinases	Peptidase C1A	Papain-like cysteine endopeptidase
				Peptidase C1A, papain C-terminal
				Cathepsin propeptide inhibitor domain (I29)
				Granulin
TRINITY DN69959 c0 g1 i24	336	332:Cathepsin L1	Peptidase C1A	Papain-like cysteine endopeptidase
				Peptidase C1A, papain C-terminal
				Cathepsin propeptide inhibitor domain (I29)
TRINITY DN69959 c0 g1 i29	314	Cysteine proteinases	Peptidase C1A	Peptidase C1A, papain C-terminal
				Cathepsin propeptide inhibitor domain (I29)
				Papain-like cysteine endopeptidase

Biology Open • Supplementary information

TRINITY DN64139 c2 g3 i05	359	6: Cathepsin S	Peptidase C1A	Peptidase C1A, papain C-terminal
				Cathepsin propeptide inhibitor domain (I29)
				Papain-like cysteine endopeptidase
JAOPJW010129046	115	4: Tissue factor pathwayinhibitor	Nonepredicted	Pancreatic trypsin inhibitor Kunitz domain
JAOPJW010158116	138	1: ATP-dependent zinc metalloprotease FtsH	Nonepredicted	Translation elongation factor EFG/EF2, domain IV
JAOPJW010002573	200	40: Serineproteaseinhibitor, putative	Nonepredicted	Pancreatic trypsin inhibitor Kunitz domain

Biology Open • Supplementary information

Table S3. *Octopus maya* proteins selected for homology modeling.

Sequence
TRINITY DN64673 c4 g1 i09
TRINITY DN64673 c4 g1 i05
TRINITY DN64673 c4 g1 i13
TRINITY DN72678 c5 g1 i02
TRINITY DN78812 c1 g1 i28
TRINITY DN78812 c1 g1 i34
TRINITY DN78812 c1 g1 i35
TRINITY DN78812 c1 g1 i16
TRINITY DN78812 c1 g1 i38
TRINITY DN78812 c1 g1 i43
TRINITY DN60096 c0 g1 i01
TRINITY DN61172 c0 g1 i03
TRINITY DN61172 c0 g1 i05
TRINITY DN76162 c1 g1 i03
TRINITY DN77537 c4 g4 i01
TRINITY DN69959 c0 g1 i24
JAOPJW010129046
JAOPJW010158116
JAOPJW010002573

Biology Open • Supplementary information

Table S4. Structural characteristics and subcellular localization of cathepsins from *Octopus maya*:

Sequence	Lengthaa	Number of predicted TMHs	Exp number of AAs in TMHs	Exp number, first 60 AAs	Total prob of N-in	POSSIBLE N-term signal sequence		
						Inside	TMhelix	Outside
TRINITY DN64673 c4 g1 i09	318	1	16.53263	16.53158	0.87629	1-6	7-24	25-318
TRINITY DN64673 c4 g1 i05	222	0	0.25739	0.25696	0.01472	-	-	1-222
TRINITY DN64673 c4 g1 i13	319	0	0.00042	0	0.00809	-	-	1-319
TRINITY DN72678 c5 g1 i02	307	1	20.557	20.09638	0.66947	1-3	4-26	27-307
TRINITY DN78812 c1 g1 i28	433	0	0.02796	0	0.00219	-	-	1-433
TRINITY DN78812 c1 g1 i34	429	0	0.03087	0	0.00241	-	-	1-429
TRINITY DN78812 c1 g1 i35	372	0	0.03552	0	0.00873	-	-	1-372
TRINITY DN78812 c1 g1 i16	345	0	0.04579	0.00331	0.01281	-	-	1-345
TRINITY DN78812 c1 g1 i38	355	0	0.03266	0	0.00403	-	-	1-355
TRINITY DN78812 c1 g1 i43	359	0	0.02966	0	0.00366	-	-	1-359
TRINITY DN60096 c0 g1 i01	293	0	2.77452	1.24932	0.07048	-	-	1-293

Biology Open • Supplementary information

TRINITY DN61172 c0 g1 i03	508	0	11.16093	11.11899	0.47234	-	-	1-508
TRINITY DN61172 c0 g1 i05	508	0	11.13698	11.12273	0.47218	-	-	1-508
TRINITY DN76162 c1 g1 i03	546	0	1.17618	1.14707	0.06032	-	-	1-546
TRINITY DN77537 c4 g4 i01	338	0	1.13962	1.1394	0.05761	-	-	1-338
TRINITY DN69959 c0 g1 i24	336	0	0.16014	0.15899	0.01034	-	-	1-336
JAOPJW010129046	115	0	0.39096	0.2953	0.12992	-	-	1-115
JAOPJW010158116	138	1	22.21533	22.21245	0.55342	38-138	15-37	1-14
JAOPJW010002573	200	0	0.0396	0.0065	0.0362	-	-	1-200

Biology Open • Supplementary information

Table S5. Homology modeling parameters obtained for *Octopus maya* cathepsins. Initial and refined values obtained in GalaxyRefine, Z-score obtained in ProSA-web. ***NC**, not calculate.

ID	RMSD	MolProbity	Clash score	Poor rotamers	Rama favored	RMSD	MolProbity	Clash score	Poor rotamers	Rama favored	Z-Score
	Refine					Initial					
TRINITY DN64673 c4 g1 i09	0.32	1.478	8.9	0.4	99.4	0	1.181	0.8	1.1	94.3	-7.46
TRINITY DN64673 c4 g1 i05	0.32	1.454	8.4	0	99.1	0	1.136	1.2	1.1	95.9	-5.96
TRINITY DN64673 c4 g1 i13	0.238	1.571	11.4	0.4	98.4	0	0.988	0.6	0	95.9	-8.04
TRINITY DN72678 c5 g1 i02	0.345	1.544	10.6	0.4	99	0	1.384	1.7	1.6	95.4	-5.7
TRINITY DN78812 c1 g1 i28	0.352	1.57	11.3	0.3	98.6	0	1.667	2.9	1.3	91.9	-8.2
TRINITY DN78812 c1 g1 i34	0.311	1.534	10.3	0.3	99.1	0	1.185	1.5	1.1	96	-8.08
TRINITY DN78812 c1 g1 i35	0.365	1.49	9.2	0.3	99.5	0	1.47	3.1	0.9	94.6	-6.91
TRINITY DN78812 c1 g1 i16	0.386	1.463	8.6	0	98.8	0	1.234	1.5	0.3	95	-7
TRINITY DN78812 c1 g1 i38	0.312	1.531	10.3	0.3	99.2	0	1.251	1.8	1.3	96.6	-7.45
TRINITY DN78812 c1 g1 i43	0.416	1.497	9.4	0.3	98.3	0	1.517	3	0.7	93.6	-7.35

Biology Open • Supplementary information

TRINITY DN60096 c0 g1 i01	0.37	1.41	7.5	0	100	0	1.069	0.5	0.8	93.8	-5.85
TRINITY DN61172 c0 g1 i03	0.455	1.51	9.7	0.7	99	0	1.587	1.3	1.7	89.7	-7.96
TRINITY DN61172 c0 g1 i05	0.465	1.493	9.3	0.7	98	0	1.531	1.7	1.2	90.1	-8.24
TRINITY DN76162 c1 g1 i03	0.361	1.62	12.9	0.6	99.1	0	1.178	2	1.1	96.9	-9.48
TRINITY DN77537 c4 g4 i01	0.332	1.515	9.8	0.3	99.1	0	1.485	1.2	2.1	93.8	-7.49
TRINITY DN69959 c0 g1 i24	0.326	1.569	11.3	0.4	99.4	0	1.565	2.1	1.4	92.5	-7.75
JAOPJW010129046	0.637	1.307	5.7	0	99.1	0	3.268	24.7	4.9	67.3	*NC
JAOPJW010158116	0.611	1.154	3.6	0.9	99.3	0	3.019	28.7	2.6	77.9	NC
JAOPJW010002573	0.525	1.367	6.7	0.6	100	0	3.418	29.7	6.3	68.7	NC

Biology Open • Supplementary information

Table S6. Crude reads and the tissues where RNA was collected

#	Traetment	Condition	Tissue	Sex	Biological replicate	Crude reads R1	Crude reads R2
1	24	PR	Oviductal gland	Male	P	GOV_H_PR_AD_24_P_F.fastq.gz	GOV_H_PR_AD_24_P_R.fastq.gz
2	24	DE	Oviductal gland	Male	P	GOV_H_DE_AD_24_P_F.fastq.gz	GOV_H_DE_AD_24_P_R.fastq.gz
3	24	PO	Oviductal gland	Male	P	GOV_H_PO_AD_24_P_F.fastq.gz	GOV_H_PO_AD_24_P_R.fastq.gz
4	RA	DE	Oviductal gland	Male	P	GOV_H_DE_AD_RA_P_F.fastq.gz	GOV_H_DE_AD_RA_P_R.fastq.gz
5	24	PR	Optical gland 1a	Male	P	GLA_H_PR_AD_24_P_F.fastq.gz	GLA_H_PR_AD_24_P_R.fastq.gz
6	24	PR	Optical gland 2a	Male	P	GLB_H_PR_AD_24_P_F.fastq.gz	GLB_H_PR_AD_24_P_R.fastq.gz
7	24	DE	Optical gland	Male	P	GLO_H_DE_AD_24_P_F.fastq.gz	GLO_H_DE_AD_24_P_R.fastq.gz
8	24	PO	Optical gland	Male	P	GLO_H_PO_AD_24_P_F.fastq.gz	GLO_H_PO_AD_24_P_R.fastq.gz
9	RA	PR	Optical gland	Male	P	GLO_H_PR_AD_RA_P_F.fastq.gz	GLO_H_PR_AD_RA_P_R.fastq.gz
10	RA	DE	Optical gland	Male	P	GLO_H_DE_AD_RA_P_F.fastq.gz	GLO_H_DE_AD_RA_P_R.fastq.gz
11	RA	PO	Optical gland	Male	P	GLO_H_PO_AD_RA_P_F.fastq.gz	GLO_H_PO_AD_RA_P_R.fastq.gz
12	24	PR	White body	Male	P	CUB_H_PR_AD_24_P_F.fastq.gz	CUB_H_PR_AD_24_P_R.fastq.gz
13	24	PO	White body	Male	P	CUB_H_PO_AD_24_P_F.fastq.gz	CUB_H_PO_AD_24_P_R.fastq.gz
14	RA	DE	White body	Male	P	CUB_H_DE_AD_RA_P_F.fastq.gz	CUB_H_DE_AD_RA_P_R.fastq.gz
15	24	PR	White body	Female	P	CUB_M_PR_AD_24_P_F.fastq.gz	CUB_M_PR_AD_24_P_R.fastq.gz
16	24	PO	White body	Female	P	CUB_M_PO_AD_24_P_F.fastq.gz	CUB_M_PO_AD_24_P_R.fastq.gz
17	30	PR	White body	Female	P	CUB_M_PR_AD_30_P_F.fastq.gz	CUB_M_PR_AD_30_P_R.fastq.gz
18	30	PO	White body	Female	P	CUB_M_PO_AD_30_P_F.fastq.gz	CUB_M_PO_AD_30_P_R.fastq.gz
19	24	PR	Optical gland	Female	P	GLO_M_PR_AD_24_P_F.fastq.gz	GLO_M_PR_AD_24_P_R.fastq.gz
20	24	PO	Optical gland	Female	P	GLO_M_PO_AD_24_P_F.fastq.gz	GLO_M_PO_AD_24_P_R.fastq.gz
21	30	PR	Optical gland	Female	P	GLO_M_PR_AD_30_P_F.fastq.gz	GLO_M_PR_AD_30_P_R.fastq.gz
22	30	PO	Optical gland	Female	P	GLO_M_PO_AD_30_P_F.fastq.gz	GLO_M_PO_AD_30_P_R.fastq.gz
23	24	PR	Optical lob	Female	P	LOP_M_PR_AD_24_P_F.fastq.gz	LOP_M_PR_AD_24_P_R.fastq.gz
24	24	PO	Optical lob	Female	P	LOP_M_PO_AD_24_P_F.fastq.gz	LOP_M_PO_AD_24_P_R.fastq.gz
25	30	PR	Optical lob	Female	P	LOP_M_PR_AD_30_P_F.fastq.gz	LOP_M_PR_AD_30_P_R.fastq.gz
26	30	PO	Optical lob	Female	P	LOP_M_PO_AD_30_P_F.fastq.gz	LOP_M_PO_AD_30_P_R.fastq.gz
27	24	PR	Testicle	Female	1	TTO_M_PR_AD_24_1_F.fastq.gz	TTO_M_PR_AD_24_1_R.fastq.gz
28	24	PR	Testicle	Female	2	TTO_M_PR_AD_24_2_F.fastq.gz	TTO_M_PR_AD_24_2_R.fastq.gz
29	24	PR	Testicle	Female	3	TTO_M_PR_AD_24_3_F.fastq.gz	TTO_M_PR_AD_24_3_R.fastq.gz
30	24	PO	Testicle	Female	1	TTO_M_PO_AD_24_1_F.fastq.gz	TTO_M_PO_AD_24_1_R.fastq.gz
31	24	PO	Testicle	Female	2	TTO_M_PO_AD_24_2_F.fastq.gz	TTO_M_PO_AD_24_2_R.fastq.gz
32	24	PO	Testicle	Female	3	TTO_M_PO_AD_24_3_F.fastq.gz	TTO_M_PO_AD_24_3_R.fastq.gz

Biology Open • Supplementary information

33	30	PR	Testicle	Female	1	TTO_M_PR_AD_30_1_F.fastq.gz	TTO_M_PR_AD_30_1_R.fastq.gz
34	30	PR	Testicle	Female	2	TTO_M_PR_AD_30_2_F.fastq.gz	TTO_M_PR_AD_30_2_R.fastq.gz
35	30	PR	Testicle	Female	3	TTO_M_PR_AD_30_3_F.fastq.gz	TTO_M_PR_AD_30_3_R.fastq.gz
36	30	PO	Testicle	Female	1	TTO_M_PO_AD_30_1_F.fastq.gz	TTO_M_PO_AD_30_1_R.fastq.gz
37	30	PO	Testicle	Female	2	TTO_M_PO_AD_30_2_F.fastq.gz	TTO_M_PO_AD_30_2_R.fastq.gz
38	30	PO	Testicle	Female	3	TTO_M_PO_AD_30_3_F.fastq.gz	TTO_M_PO_AD_30_3_R.fastq.gz
39	24	PR	Optical lob	Male	1	LOP_H_PR_AD_24_1_F.fastq.gz	LOP_H_PR_AD_24_1_R.fastq.gz
40	24	PR	Optical lob	Male	2	LOP_H_PR_AD_24_2_F.fastq.gz	LOP_H_PR_AD_24_2_R.fastq.gz
41	24	PR	Optical lob	Male	3	LOP_H_PR_AD_24_3_F.fastq.gz	LOP_H_PR_AD_24_3_R.fastq.gz
42	24	DE	Optical lob	Male	1	LOP_H_DE_AD_24_1_F.fastq.gz	LOP_H_DE_AD_24_1_R.fastq.gz
43	24	DE	Optical lob	Male	2	LOP_H_DE_AD_24_2_F.fastq.gz	LOP_H_DE_AD_24_2_R.fastq.gz
44	24	DE	Optical lob	Male	3	LOP_H_DE_AD_24_3_F.fastq.gz	LOP_H_DE_AD_24_3_R.fastq.gz
45	24	PO	Optical lob	Male	1	LOP_H_PO_AD_24_1_F.fastq.gz	LOP_H_PO_AD_24_1_R.fastq.gz
46	24	PO	Optical lob	Male	2	LOP_M_PO_AD_24_2_F.fastq.gz	LOP_M_PO_AD_24_2_R.fastq.gz

**Condition**	**key**
pre-spawning	PS
Post-spawning	PTS
spawning	SP

P= pooled

Biology Open • Supplementary information
